# LAMP5 in presynaptic inhibitory terminals in the hindbrain and spinal cord: a role in startle response and auditory processing

**DOI:** 10.1186/s13041-019-0437-4

**Published:** 2019-03-12

**Authors:** Michinori Koebis, Shinji Urata, Yo Shinoda, Shigeo Okabe, Tatsuya Yamasoba, Kazuki Nakao, Atsu Aiba, Teiichi Furuichi

**Affiliations:** 1 Laboratory of Animal Resources, Center for Disease Biology and Integrative Medicine, Graduate School of Medicine, The University of Tokyo, Tokyo, 113-0033 Japan; 2 Department of Otolaryngology, Faculty of Medicine, The University of Tokyo, Tokyo, 113-8655 Japan; 3 Department of Environmental Health, School of Pharmacy, Tokyo University of Pharmacy and Life Sciences, Tokyo, 192-0392 Japan; 4 Department of Cellular Neurobiology, Graduate School of Medicine, The University of Tokyo, Tokyo, 113-0033 Japan; 5 Department of Applied Biological Sciences, Tokyo University of Science, Chiba, 278-8510 Japan

**Keywords:** LAMP5, Startle response, Prepulse inhibition, Inhibitory synaptic terminals, LSO, VGAT

## Abstract

Lysosome-associated membrane protein 5 (LAMP5) is a mammalian ortholog of the *Caenorhabditis elegans* protein, UNC-46, which functions as a sorting factor to localize the vesicular GABA transporter UNC-47 to synaptic vesicles. In the mouse forebrain, LAMP5 is expressed in a subpopulation of GABAergic neurons in the olfactory bulb and the striato-nigral system, where it is required for fine-tuning of GABAergic synaptic transmission. Here we focus on the prominent expression of LAMP5 in the brainstem and spinal cord and suggest a role for LAMP5 in these brain regions. LAMP5 was highly expressed in several brainstem nuclei involved with auditory processing including the cochlear nuclei, the superior olivary complex, nuclei of the lateral lemniscus and grey matter in the spinal cord. It was localized exclusively in inhibitory synaptic terminals, as has been reported in the forebrain. In the absence of LAMP5, localization of the vesicular inhibitory amino acid transporter (VIAAT) was unaltered in the lateral superior olive and the ventral cochlear nuclei, arguing against a conserved role for LAMP5 in trafficking VIAAT. *Lamp5* knockout mice showed no overt behavioral abnormality but an increased startle response to auditory and tactile stimuli. In addition, LAMP5 deficiency led to a larger intensity-dependent increase of wave I, II and V peak amplitude of auditory brainstem response. Our results indicate that LAMP5 plays a pivotal role in sensorimotor processing in the brainstem and spinal cord.

**Electronic supplementary material:**

The online version of this article (10.1186/s13041-019-0437-4) contains supplementary material, which is available to authorized users.

## Introduction

Membrane trafficking is important for the generation of specialized cell structures and functions. In neurons, formation of polarized cell morphology requires long-distance transport of intracellular membrane organelles and membrane proteins to axons and dendrites [1–6]. In presynaptic boutons and postsynaptic dendritic spines, exocytotic and endocytotic membrane trafficking regulates efficacies of synaptic neurotransmission; e.g., regulation of neurotransmitter release probability by exocytosis of synaptic vesicles [7–11] and changes in the postsynaptic response by exo/endocytosis of neurotransmitter receptors [12–14]. In addition to ubiquitously expressed molecules that regulate general membrane trafficking, there are a variety of neuron-specific molecules that exert critical roles in neuronal structure and function [15, 16].

We recently developed a transcriptome database for various developmental stages of the mouse brain (BrainTx) and have identified several genes whose expression is brain specific and developmentally regulated [17, 18]. A gene encoding lysosome-associated membrane protein 5 (LAMP5) is one such gene that shows distinctive mRNA expression patterns during postnatal stages. Although LAMP5 belongs to the LAMP family based on structural similarity in the LAMP domain, it is different from other LAMP proteins in terms of tissue-specific expression. For example, LAMP5 is almost brain-specific, whereas LAMP1 and LAMP2 are expressed in cells of many tissues besides brain [19]. Moreover, in cortical pyramidal neurons, LAMP5 is mainly accumulated in distinct intracellular vesicles, which do not contain any known markers of classical intracellular transport pathways, and is not targeted to the late endosomal/lysosomal compartments [20] in which LAMP1 and LAMP2 are primarily localized. Thus, LAMP5 is expected to play a different role from that of LAMP1 and LAMP2 and to contribute to the unique function of the central nervous system (CNS); however, the distribution and function of the protein in the mammalian CNS is not well characterized.

A recent study investigated LAMP5 expression in the mouse forebrain and revealed that it is expressed in subpopulations of GABAergic neurons in the striato-nigral system and the olfactory bulb [21]. The protein is localized to synaptic terminals, where it overlaps with the vesicular GABA transporter or vesicular inhibitory amino acid transporter (VGAT/VIAAT), and deficiency of LAMP5 leads to altered pre-synaptic plasticity in striatopallidal synapses. Interestingly, UNC-46, a LAMP5 ortholog in *Caenorhabditis elegans* is also localized in the presynaptic terminals of all 26 GABAergic neurons [22]. It is essential for the axonal transport of VIAAT; the *unc-46* mutant has disturbed GABA neurotransmission and motor coordination, suggesting a conserved role of LAMP5 as a VIAAT trafficking chaperon in the mammalian brain. However, LAMP5-deficiency in mice does not affect the subcellular localization of VIAAT in striatal neurons, posing the possibility that LAMP5 may be involved in GABA neurotransmission in a different manner in the mammalian brain [21].

There are many inhibitory neurons in the hindbrain and spinal cord of the mammalian CNS, but the expression and the subcellular localization of LAMP5 in these regions have not been reported. Therefore, in the present study, we addressed whether LAMP5 is localized in the inhibitory synaptic terminals and whether it is involved with the axonal transport of VIAAT in the brainstem and the spinal cord. We found a prominent expression of LAMP5 in these regions and, as in the forebrain, LAMP5 was localized in the synaptic terminals of the subpopulation of inhibitory neurons and was not essential for the proper localization of VIAAT in the brainstem. We further investigated the physiological function of LAMP5 by generating LAMP5-deficient mice. Interestingly, LAMP5 deficiency led to a remarkable increase in startle response and in auditory brainstem response especially at higher sound pressure level. Our results indicate a role of LAMP5 in senserimotor processing in the hindbrain and spinal cord.

## Results

### Differential expression patterns of LAMP5 protein in the postnatal mouse CNS

**Fig. 1:**
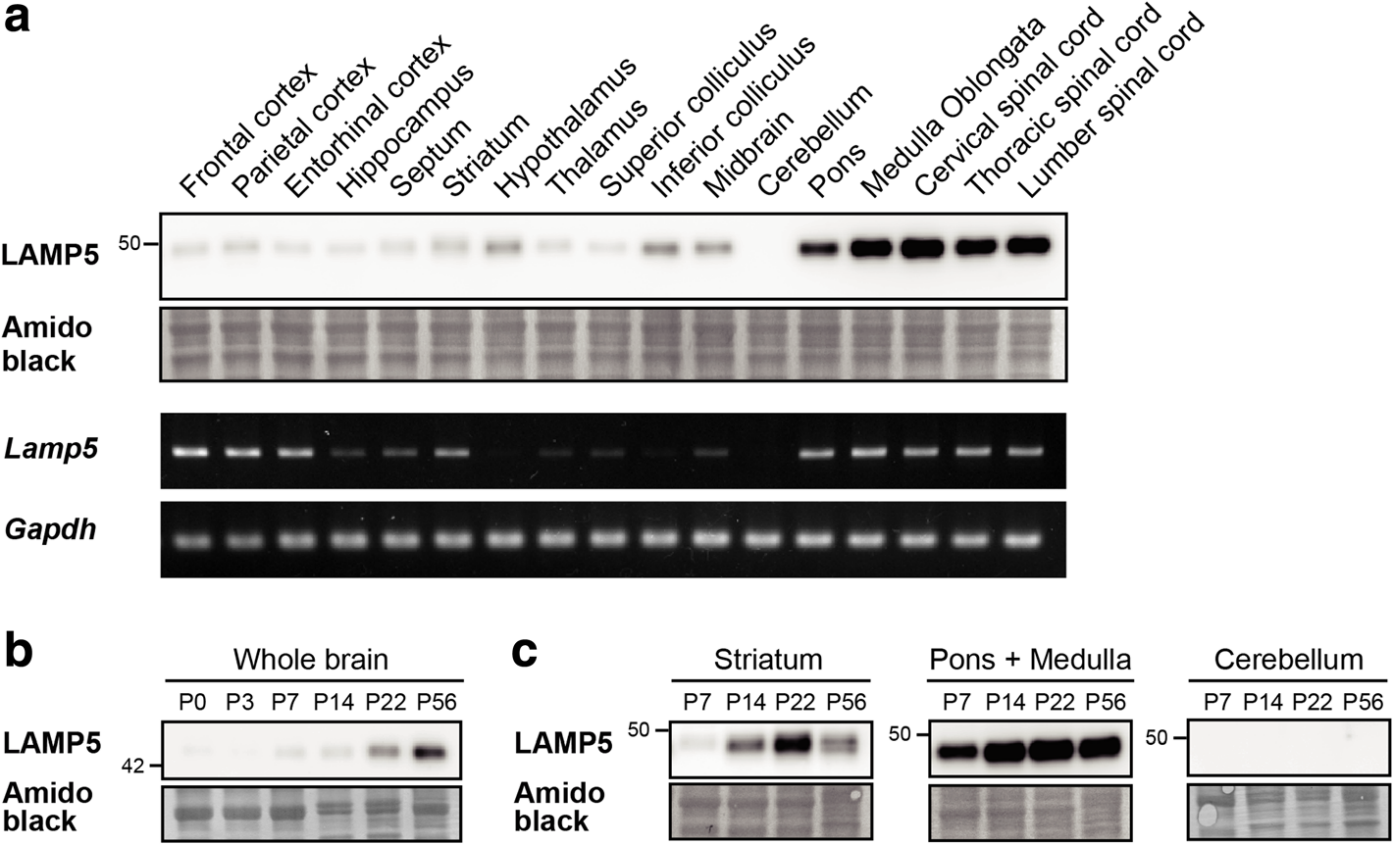
Spatiotemporal expression of LAMP5 protein in various brain regions. **a** Spatial distribution of LAMP5 protein and *Lamp5* mRNA at P56. Total protein lysates and total RNA of indicated regions were subjected to western blotting (upper panel) and RT-PCR analyses (lower panel), respectively. Midbrain was the remaining of midbrain after the superior and inferior colliculi were removed. Amido black staining and *Gapdh* are shown as loading controls. **b** and **c** Developmental expression of LAMP5. LAMP5 expression in whole brains at P0, P3, P7, P14, P22 and P56 (**b**), and in the striatum, cerebellum, pons and medulla oblongata at P7, P14, P22 and P56 was analyzed (**c**)

We investigated the spatiotemporal expression profile of LAMP5 in the mouse CNS. We first analyzed 18 different regions of the adult mouse CNS, including the hindbrain regions and the spinal cord, and found that LAMP5 protein was expressed throughout the CNS with prominent expression in the pons, the medulla oblongata and the spinal cord (Fig. 1a). Moderate expression was observed in the inferior colliculus and the striatum, while expression was below the limit of detection in the cerebellum. LAMP5 expression was also observed in hypothalamus lysate; however, this was possibly because of incorporation of flanking regions, such as the ventral pallidum and the substantia nigra, because subsequent immunohistochemical analysis revealed that the flanking regions expressed LAMP5 strongly whereas the hypothalamus itself did not [21] (see below). Interestingly, the expression profile of *Lamp5* mRNA was different from that of LAMP5 protein (lower panels in Fig. 1a). On one hand, a striking difference was observed in the cerebral cortex, where the transcript was highly expressed but the protein was barely detectable. On the other hand, the transcript was hardly detected in certain regions like the inferior colliculus and the hypothalamus where the protein was present, indicating that the protein was translated elsewhere and transported to the regions via axonal projections.

We also analyzed LAMP5 expression during postnatal development at postnatal day 3 (P3), P7, P14, P22 and P56 in whole brain (Fig. 1b) and in several brain regions (Fig. 1c). Immunoblotting of whole brain samples revealed that LAMP5 expression increased drastically in the period between weaning and young adulthood (Fig. 1b). However, the expression levels of LAMP5 in each brain region showed regional specificity during development (Fig. 1c). In the striatum, the expression level of LAMP5 increased during postnatal development and reached a peak at P22 (juvenile stage). High expression was already detected at P7 in the pons and medulla, and reached a plateau at P14 that was maintained thereafter. In contrast, expression in cerebellar samples could not be detected at any postnatal stages. These results showed that LAMP5 is widely but specifically expressed in numerous brain regions during postnatal development with prominent expression in the hindbrain and spinal cord.

### Restricted distribution of LAMP5 protein in the brain stem

**Fig. 2:**
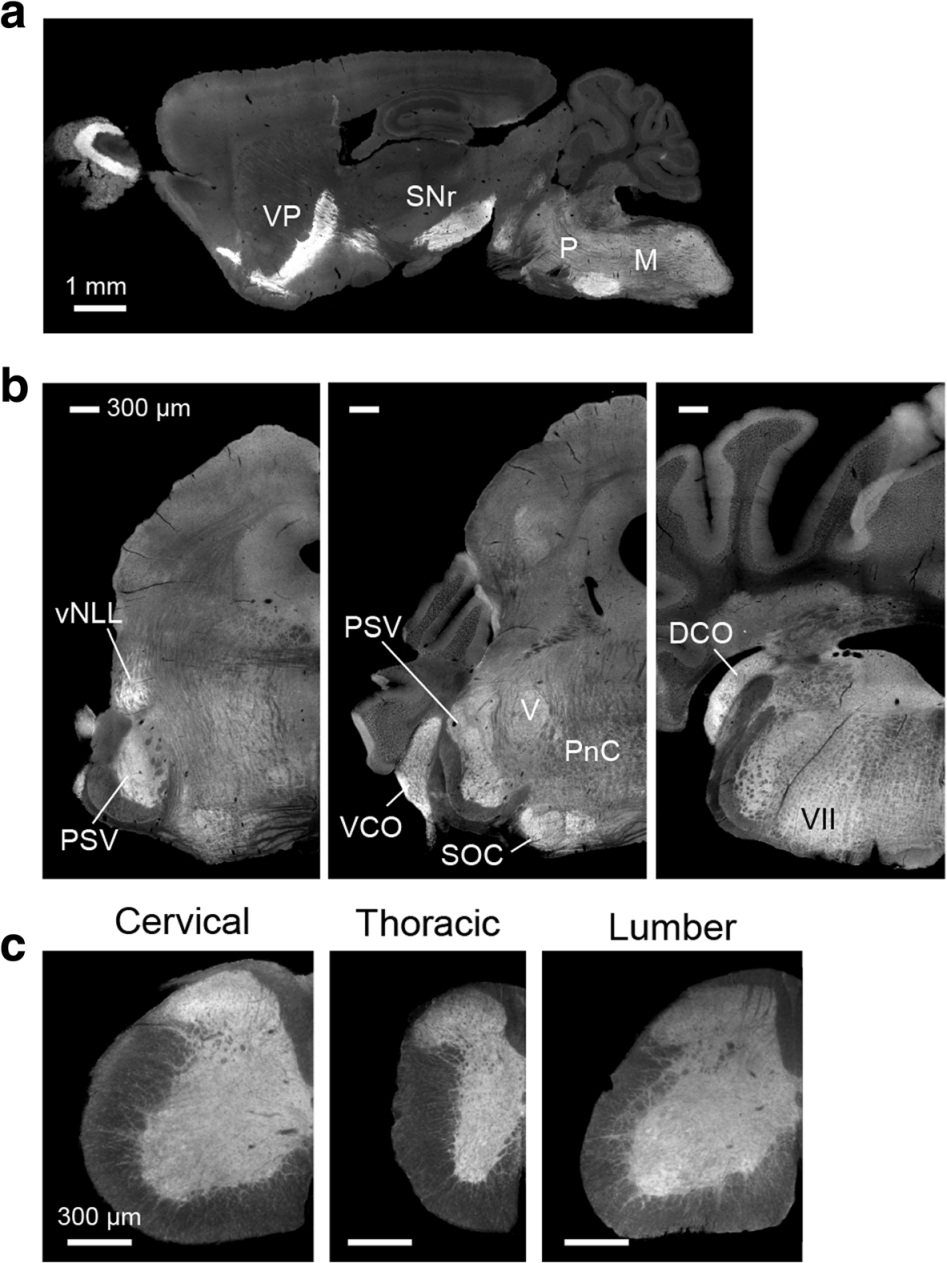
LAMP5 immunoreactivity in the brain and spinal cord. A sagittal section of an adult mouse brain (**a**), and coronal sections of an adult brainstem (**b**) and spinal cord (**c**) were immunostained against LAMP5. *DCO* dorsal cochlear nucleus; *M* medulla oblongata; *P* pons, *PnC* Caudal pontine reticular formation, *PSV* principal sensory nucleus of the trigeminal, *SOC* superior olivary complex, *SNr* substantia nigra par reticulata, *VCO* ventral cochlear nucleus, *vNLL* ventral nucleus of lateral lemniscus, *VP* ventral pallidum. Scale bars: **a**, 1 mm; **b**, 300 μm; **c**, 300 μm

Next, we investigated the distribution of LAMP5 in the adult brain and spinal cord by immunohistochemistry (Fig. 2). Consistent with the immunoblot results, strong immunoreactivity was observed in the pons and the medulla oblongata. The protein was also present in the ventral pallidum (VP) and the substantia nigra pars reticulata (SNr) as previously reported [21] (Fig. 2a).

In coronal hindbrain sections LAMP5 was broadly distributed in the pons and the medulla oblongata with marked expression in the sensory-related nuclei of the pons and cochlear nuclei (Fig. 2b): namely, the ventral part of the nucleus of lateral lemniscus (vNLL), the principal sensory nucleus of the trigeminal nerve (PSV), the superior olivary complex (SOC) and the ventral and dorsal parts of the cochlear nucleus (VCO and DCO).

In the spinal cord, LAMP5 immunoreactivity was present throughout the dorsal, lateral and ventral horns of the gray matter and also in nerve fibers within the dorsal, lateral and ventral columns of the white matter (Fig. 2c). Similar distribution patterns were observed in the cervical, thoracic and lumber regions of the spinal cord.

### LAMP5 is expressed in inhibitory projection neurons in the brainstem

**Fig. 3:**
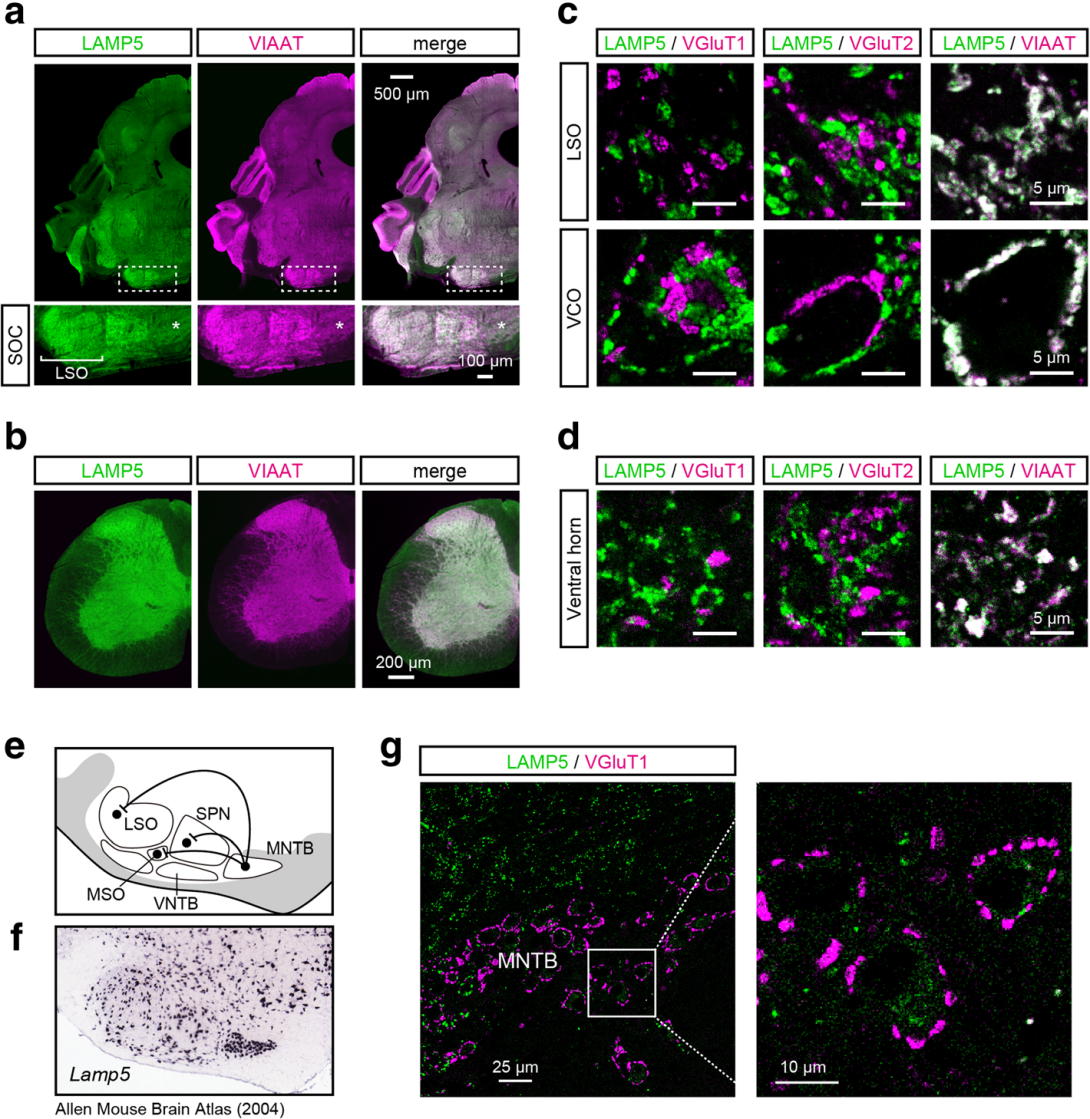
LAMP5 is localized in the inhibitory synaptic terminals. **a** LAMP5 and VIAAT expression in the adult brainstem. Lower panels: higher-magnification images of the superior olivary complex (SOC) at the positions indicated in the top images (rectangles with white dashed lines). Scale bars: upper, 500 μm; lower, 100 μm. **b** LAMP5 and VIAAT expression in the cervical spinal cord. Scale bars, 200 μm. **c** and **d** Confocal images of the lateral superior olive (LSO) and ventral cochlear nucleus (VCO) regions (**c**) and the ventral horn of the cervical spinal cord (**d**). LAMP5 was double-stained with VGluT1, VGluT2 or VIAAT. Scale bars, 5 μm. **e** Schematic diagram of the SOC nuclei. The medial nucleus of the trapezoid body (MNTB) neurons send inhibitory projection to the LSO, medial superior olive (MSO) and superior paraolivary nucleus (PSV) neurons. **f** Distribution of *Lamp5*-expressing neurons in the SOC. In situ hybridization data is from the Allen Mouse Brain Atlas (Experiment No. 70927827) [23]. **g** LAMP5 expression in MNTB neurons. The cell body of an MNTB neuron can be identified because excitatory synaptic terminals surround it. Right panel: Higher-magnification image of the MNTB neurons indicated by the white square in the left image. Scale bars: left, 25 μm; right, 10 μm

Next, we examined whether LAMP5 was localized to inhibitory synaptic terminals in the hindbrain and the spinal cord. When we double-stained inhibitory axon termini with anti-LAMP5 and anti-VIAAT antibodies, the distributions of LAMP5 and VIAAT were highly similar in the hindbrain and spinal cord (Fig. 3a–b). For example, both VIAAT and LAMP5 showed a strong gradient across the lateral superior olive (LSO) with higher immunoreactivity in the lateral limb of this nucleus (lower panels in Fig. 3a). At a higher magnification, we found that LAMP5 was specifically co-localized with VIAAT and segregated from either vesicular glutamate transporter 1 (VGluT1)- or VGluT2-positive synaptic boutons (Fig. 3c). The co-localization of LAMP5 and VIAAT was also observed in the VCO, the vNLL, the PSV, the caudal pontine reticular formation (PnC) and the ventral and dorsal horn of the spinal cord (Fig. 3c–d and data not shown).

The LSO neurons receive major glycinergic input from principal neurons in the medial nucleus of the trapezoid body (MNTB) (Fig. 3e). According to the Allen Mouse Brain Atlas [23, 24], MNTB neurons express *Lamp5* mRNA at a high level (Fig. 3f) but the MNTB itself did not show a strong immunoreactivity against LAMP5 (Fig. 3a, asterisk) and only weak immunoreactivity was detected in the cytoplasm of MNTB principal neurons (Fig. 3g). This suggests that LAMP5 expressed in MNTB neurons was transported to their synaptic terminals located in the LSO.

### Generation of a *Lamp5*-deficient mouse

**Fig. 4:**
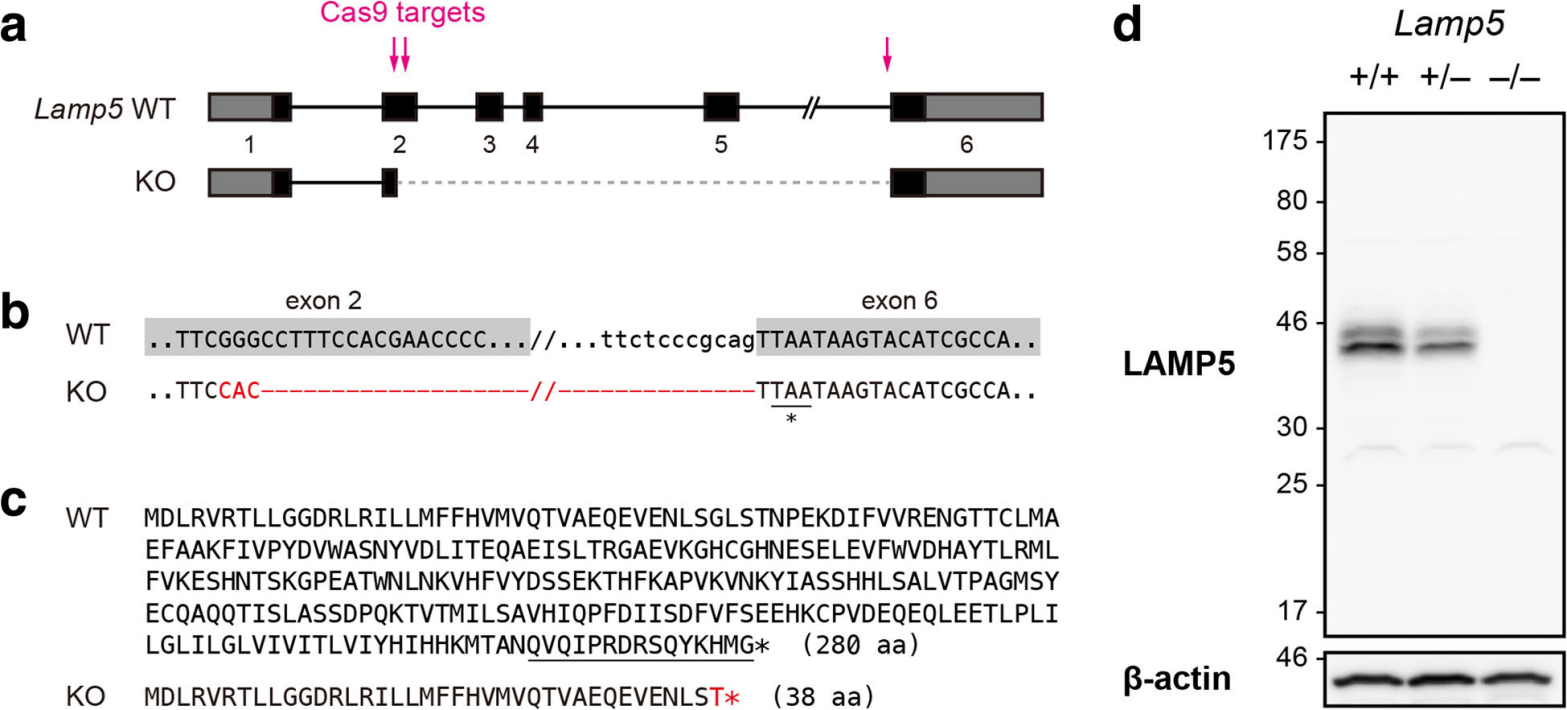
Generation of a *Lamp5* KO mouse. **a** Schematic diagram of the wild-type (WT) and *Lamp5* KO alleles. Arrows, CRISPR/Cas9 target sites; gray box, untranslated region; black box, coding region; solid line, intron; dashed line, deleted region in the KO allele. Exon 2 is fused with exon 6. **b** DNA sequences of the WT and KO alleles. The mutated sequence is shown in red. A termination codon appears at the beginning of exon 6 (underlined). **c** Amino acid sequences of proteins encoded by the WT and KO alleles. The epitope used for primary antibody production is underlined. **d** LAMP5 protein expression in whole brain lysates of WT (+/+), heterozygous and homozygous *Lamp5* KO (+/− and −/−) mice. β-actin served as a loading control

To reveal molecular and physiological functions of LAMP5, we generated a *Lamp5* mutant mouse line using the CRISPR/Cas9 system. We used three guide RNAs targeting either exon 2 or the 3′ splice site of intron 5 of the *Lamp5* gene and obtained a mutant mouse carrying a deletion spanning from the middle of exon 2 to the 3′ end of intron 5 with a 3-base insertion (Fig. 4a and b). The mutation resulted in the in-frame fusion of the 5′ half of exon 2 and the last exon, and the loss of most of the LAMP5 protein other than the signal peptide. Thus the polypeptide encoded by the mutant allele is predicted to be non-functional (Fig. 4c). The absence of the full-length LAMP5 protein was confirmed by western blotting of whole brain lysates from wild type, and heterozygous and homozygous *Lamp5* mutants (Fig. 4d). Hereafter, we call the homozygous mutant *Lamp5* KO.

### Involvement of LAMP5 in axonal transport of VIAAT

**Fig. 5:**
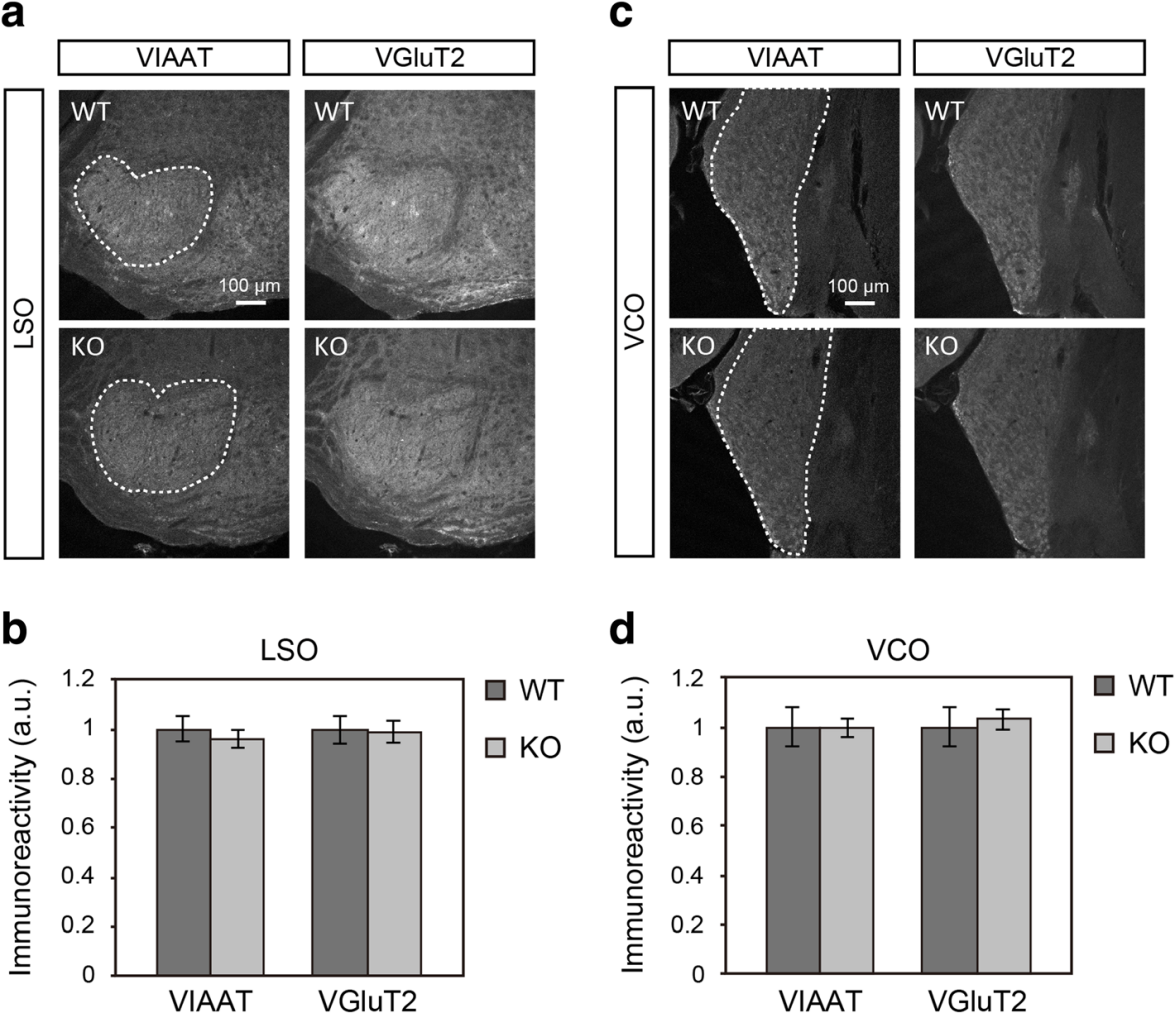
VIAAT and VGluT2 in the LSO and VCO in *Lamp5* KO mice. **a** VIAAT and VGluT2 immuoreactivities in the LSO of WT and KO sections. **b** Quantitative analysis of VIAAT and VGluT2 immunoreactivities in the LSO (indicated by dashed lines in **a**). **c** VIAAT and VGluT2 immuoreactivities in the VCO. **d** Quantitative analysis of VIAAT and VGluT2 immunoreactivities in the VCO (indicated by dashed lines in **c**). Two-tailed Welch’s t-test revealed no significant difference between the genotypes, either in the LSO or VCO (*p* > 0.05, *n* = 4 mice for each genotype). Error bars represent SEM

Using *Lamp5* KO mice, we examined whether LAMP5 was involved with the axonal transport of VIAAT. LAMP5-expressing MNTB neurons provide major glycinergic input to the LSO neurons; therefore, we investigated VIAAT expression in the LSO. The immunoreactivity for VIAAT and the ratio of VIAAT to VGluT2 were not altered in KO animals (Fig. 5a–b, and data not shown). We also investigated the VCO and failed to detect any change in VIAAT expression in *Lamp5* KO mice (Fig. 5c–d). These results indicate that LAMP5 is not essential for the axonal transport of VIAAT in these nuclei.

### Exaggerated startle response in *Lamp5* KO mice

**Fig. 6:**
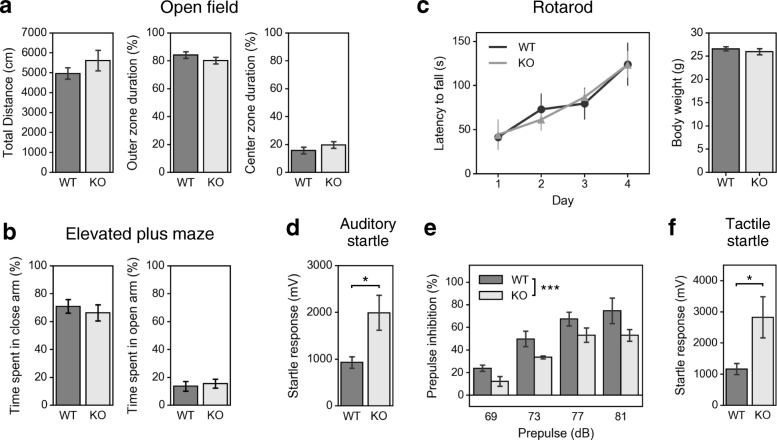
Behavioral analysis of *Lamp5* KO mice. **a** Open field test. There was no significant difference between genotypes in the total distance travelled or in the time spent in the outer or inner zones (two-tailed Welch’s t-test, *n* = 8 mice for each genotype). **b** Elevated plus maze test. There was no significant difference between genotypes in time spent in the open arm or the closed arm (two-tailed Welch’s t-test, *n* = 8 mice for each genotype). **c** Rotarod test. Two-way repeated-measures ANOVA (genotype × time) revealed a significant main effect of time (F_(3,42)_ = 9.458, *p* < 0.0001), a non significant effect of genotype (F_(1,15)_ = 2.51, *p* > 0.05) and a non significant interaction between factors (F_(3,42)_ = 0.1307, *p* = 0.9413). Body weight after the fourth trial was not different between genotypes (two-tailed Welch’s t-test, *n* = 8 mice for each genotype). **d** Startle response to 120 dB white noise stimuli. The response was larger in KO mice (*p* < 0.05, Two-tailed Welch’s t-test, *n* = 5 mice for each genotype). **e** Percent inhibition of auditory startle response by prepulse stimulation. Two-way repeated-measures ANOVA (genotype × prepulse) revealed a significant effect of prepulse intensity (F_(3,32)_ = 23.0, *p* < 10^− 7^) and of genotype (F_(1,32)_ = 13.2, *p* < 0.001) but no significant interaction between factors (F_(3,32)_ = 2.90, *p* > 0.05). **f** Startle response to air-puff stimuli. The response was larger in KO mice (*p* < 0.05, Two-tailed Welch’s t-test, *n* = 8 mice for each genotype). * *p* < 0.05, *** *p* < 0.001. Error bars indicate SEM

**Fig. 7:**
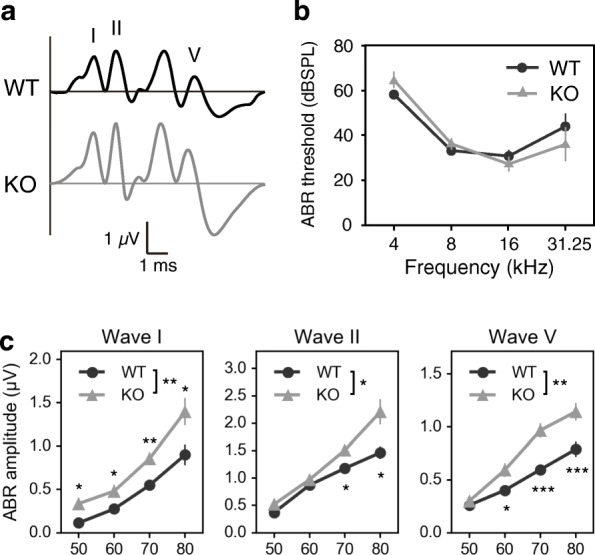
Auditory brainstem response (ABR) of *Lamp5* KO mice. **a** Representative ABR waveform to 12 kHz tone-burst stimuli recorded from wild type and a *Lamp5* KO. **b** ABR threshold. Two-way repeated-measures ANOVA (genotype × frequency) revealed a significant main effect of frequency (F_(3,36)_ = 57.68, *p* < 0.0001), a non significant effect of genotype (F_(1,12)_ = 0.0124, *p* = 0.9133) and a significant interaction between factors (F_(3,36)_ = 2.921, *p* = 0.0471). Post-hoc Welch’s t-test revealed no significant difference between genotypes at either frequency (*n* = 7 for each genotype). **c** Amplitude of ABR waves at 16 kHz. The waves were indicated in **a**. Two-way repeated-measures ANOVA (genotype × sound intensity) and post-hoc Welch’s t-test were performed for each wave. A significant main effect of genotype and a significant interaction between factors were present in wave I (F_(1,12)_ = 10.15, *p* = 0.0078; F_(3,36)_ = 2.301, *p* = 0.0936, respectively), wave II (F_(1,12)_ = 8.977, *p* = 0.0111; F_(3,36)_ = 4.062, *p* = 0.0139, respectively) and wave V (F_(1,12)_ = 16.19, *p* = 0.0017; F_(3,36)_ = 5.341, *p* = 0.0038, respectively). * *p* < 0.05, ** *p* < 0.01, *** *p* < 0.001. Error bars indicate SEM

Next, we performed a series of behavioral tests including tests that evaluate brainstem- and spinal cord-related functions. Open field, elevated plus maze and rotarod tests showed that *Lamp5* KO mice have no overt abnormalities in locomotor activity, anxiety-related behavior, motor coordination and motor learning (Fig. 6a–c). Interestingly, however, we found that *Lamp5* KO mice showed increased startle response to unexpected loud noise (Fig. 6d). The mutants also showed decreased prepulse inhibition (PPI) of the acoustic startle reflex (Fig. 6e), indicating that LAMP5 deficiency led to abnormal sensorimotor processing. Because LAMP5 was expressed in the inhibitory synaptic terminals in the cochlear nuclei, the SOC and the vNLL, all of which are involved with auditory processing, we assumed that the increased startle reflex in *Lamp5* KO mice might result from increased excitability of the brainstem auditory circuit. We therefore analyzed auditory brainstem response (ABR) to tone-burst stimuli at various frequencies (4, 8, 16 and 31.25 kHz) (Fig. 7). ABR threshold was not different between the genotypes (Fig. 7b), but the amplitude of ABR appeared to be larger in KO animals (Fig. 7a). When we analyzed the peak amplitude of ABR waves at 16 kHz, wave I, II and V were significantly larger especially at the higher sound pressure level. Meanwhile, a tactile stimulus also elicited an increased startle response in the KO mice (Fig. 6f), indicating that there was no stimulus specificity in the exaggeration of the startle response in KO mice. Taken together, knockout of the *Lamp5* gene led to an increased startle response and smaller PPI as well as an increased ABR, indicating that LAMP5 plays an essential role in regulating brainstem- and spinal cord-mediated sensorimotor and auditory processing ability. These findings are consistent with the immunohistochemistry findings.

## Discussion

In the present study, we characterized the spatial and temporal distribution of LAMP5 in the mouse hindbrain and spinal cord. We found that LAMP5 was expressed in the pons and medulla oblongata at relatively high levels by P7. According to the Allen Developing Mouse Brain Atlas [25], expression of *Lamp5* mRNA can be observed in the hindbrain and spinal cord as early as at embryonic day 11.5 (E11.5) and then spreads to the midbrain and forebrain regions after birth [23], as also shown in this study. In the spinal cord, synaptic contact occurs on motor neurons at E11.5 and the first synaptic activity can be recorded the following day [26–30], whereas synaptogenesis occurs postnatally in the forebrain and peaks in the juvenile stage [31]. Thus, the temporal transition of LAMP5 expression was consistent with the synaptic formation in the striatum, pons and medulla.

Our immunohistochamical analysis showed that LAMP5 was localized exclusively in inhibitory synaptic terminals in the pons and spinal cord, suggesting its role in inhibitory neurotransmission. Two studies have reported that LAMP5 and its ortholog function in GABAergic nerve terminals [21, 22]. One showed that in *C. elegans*, the LAMP5 ortholog, UNC-46, is expressed in every GABAergic motor neuron and acts as a sorting chaperone that localizes VIAAT to synaptic terminals [22]. In *unc-46* mutants, frequency of miniature currents is severely reduced possibly due to a reduced number of vesicles that harbored the transporter. The other study showed that in mammals, LAMP5 is localized in inhibitory synaptic terminals of granule cells of the olfactory bulb and in striatal neurons [21]; however, the study also showed that LAMP5 is not essential for correct localization of VIAAT in these neurons, arguing against the conserved role of axonal trafficking of the transporter. In the present study, we showed that VIAAT immunoreactivity was not altered in the LSO or VCO of LAMP5-deficient mice, indicating that LAMP5 has little to do with terminal localization of VIAAT in the brainstem. Nevertheless, LAMP5-deficiency has a complex impact on the striatopallidal synapse function: the frequency of activity-independent currents is increased while the evoked release probability is decreased as indicated by a strong paired-pulse facilitation of inhibitory postsynaptic currents [21]. Although it is yet unknown how LAMP5 affects presynaptic release machinery, the study suggested the importance of LAMP5 on fine-tuning of GABAergic neurotransmission. We speculate that LAMP5 achieves its function by regulating turnover of some membrane proteins or vesicles, because a study of cortical neurons that showed LAMP5 was subjected to endocytosis and localized in uncharacterized early endocytic organelles [20]. Taken together, these results suggest that LAMP5-deficiency can lead to a compromised neurotransmission of inhibitory synapses in the hindbrain and spinal cord.

LAMP5 deficiency did not cause any overt behavioral abnormality but specifically led to increased startle responses to both auditory and tactile stimuli. Interestingly, dysfunction of glycinergic neurotransmission, which is a major source of synaptic inhibition in the hindbrain and spinal cord, is known to cause exaggerated startle response. For example, the mouse mutant *spasmodic*, which carries a mutation in the gene encoding the glycine receptor subunit alpha 1 (*Glra1*) that decreases its ligand binding affinity, presents massive tremor and exaggerated startle response to sound and touch three weeks after birth [32–34]. Similar phenotypes are caused by mutations in genes encoding glycine receptor subunit beta (*Glrb*) [35, 36], glycine transporter 2 (*Slc6a5*) [37] or gephyrin [38], which is required in glycine receptor clustering [39]. Therefore, one possible explanation of the increased startle response is that LAMP5 deficiency led to dysfunctional glycinergic neurotransmission in the brainstem and spinal cord. Auditory and tactile stimuli are conveyed via the auditory and trigeminal sensory pathway, respectively, to giant neurons in the PnC. The neurons send direct projections to motor neurons that innervate facial and skeletal muscles and elicit movement of startle reflex. LAMP5 can potentially affect startle response either upstream or downstream from the PnC because the protein was expressed both in nuclei composing the auditory and trigeminal pathways (e.g., VCO and PSV) and in the ventral horn of spinal cord where somata of motor neurons exist. The startle response can also be potentiated by fear and anxiety [40]; however, because our behavioral analysis showed that LAMP5 deficiency did not affect emotional behavior, and a study from another group showed that it even decreases anxiety [21], it is unlikely that the startle response was potentiated by the animals’ anxious state.

The normal ABR threshold of LAMP5-deficient mice indicates that function of the cochlear hair cell is conserved. However, the mutant mice showed a larger increase in peak amplitude of waves I, II and V than wild type controls as the sound pressure level of auditory stimuli increased. The larger amplitude of wave I indicates auditory input evokes greater neuronal activity already at the cochlear spiral ganglion. The function and expression of LAMP5 in the cochlea has yet to be shown, but the protein can possibly regulate the cochlear output via SOC neurons because the SOC gives rise to the olivocochlear system that is involved with a descending control of the cochlea [41]. Especially, the medial olivocochlear system (MOCS) is a strong candidate that may lead the altered ABR in *Lamp5* KO animals. Several studies demonstrated that electrical stimulation of the MOCS decreases activity of the auditory nerve by inhibiting the active mechanical amplification process of the outer hair cells [42–45]. The MOCS activity depends on sound pressure level of input [46]; therefore, a compromised function of the MOCS will increase auditory nerve responses especially when a loud auditory stimulus is applied. Because the input/output function of ABR waves was increased in *Lamp5* KO mice, we assume that LAMP5 deficiency brings about functional deficits in the MOCS thereby enhancing neural activity of the cochlear nerve at higher intensity of sound input. The MOCS stems from large multipolar neurons that reside in the ventral nucleus of the trapezoid body (VNTB), where neurons highly express *Lamp5* mRNA according to Allen Mouse Brain Atlas. Thus it is likely that LAMP5 functions in the MOCS. Thus elevated response to auditory stimuli is considered to contribute at least in part to the potentiated auditory startle response.

The present study showed LAMP5 to be expressed widely in the inhibitory synaptic terminals of the pons, medulla oblongata and spinal cord and indicates a role in the acoustic and tactile startle reflex. Our results exclude the possibility that the exaggerated startle response in *Lamp5*-deficient mice was caused by decreased axonal trafficking of VIAAT. Further studies will elucidate the detailed physiological properties of LAMP5 in this reflex pathway.

## Methods

### Animals

C57BL/6J mice were purchased from CLEA Japan, Tokyo, Japan. Recipient ICR mice were purchased from Charles River Laboratories JAPAN, Kanagawa, Japan. All animals were treated according to the recommendations and protocols approved by the Animal Care and Use Committee of Tokyo University of Science and by the Animal Welfare Committee of the University of Tokyo. Animals were housed in an environment with a 12-h/12-h light/dark cycle (daytime 8:00–20:00) with controlled temperature (23 ± 2 °C) and humidity (55 ± 10%) and ad libitum access to food and water.

### Cas9 mRNA and sgRNA preparation

A plasmid template for in vitro transcription of *Cas9* mRNA was kindly provided by Dr. T. Mashimo (Kyoto University). The plasmid encodes a Cas9 protein flanked by SV40 nuclear localization signals with a 90-bp poly(A) tail in the 3′-untranslated region, which facilitates its translation in zygotes [47]. *Cas9* mRNA was transcribed using a MessageMAX T7 ARCA-Capped Message Transcription Kit (CELLSCRIPT, Madison, WI, USA), polyadenylated with an A-Plus Poly(A) Polymerase Tailing Kit (CELL SCRIPT) and purified using a MEGAclear Kit (Life Technologies). Three single guide RNAs (sgRNAs) were designed to target either exon 2 or 6 of the *Lamp5* gene (Fig. 4). Their protospacer sequences with PAM (underlined) were as follows: CTTTCTCGGGGTTCGTGGAAAGG, AAATGGGACGACGTGTCTCATGG and GCGATGTACTTATTAACTGCGGG. These protospacers were cloned into *Bsa*I-digested pDR274 vector (Addgene ID #42250) and the resulting plasmids served as templates for in vitro transcription. The sgRNAs were transcribed using a MEGAshortscript T7 Transcription Kit (Thermo Fisher Scientific K.K., Tokyo, Japan) and purified using a mini Quick Spin Column (Roche Diagnostics K.K., Tokyo, Japan). All procedures were performed according to the manufacturers’ instructions.

### Embryo collection and electroporation

*Cas9* mRNA and sgRNA were delivered by electroporation to C57BL/6J embryos at the pronuclear stage as described previously with slight modifications [48]. Briefly, the embryos were washed three times with Opti-MEM I (Thermo Fisher Scientific) supplemented with 0.1% polyvinylalchol (PVA) and once with 0.1% PVA-Opti MEM I containing *Cas9* mRNA (400 ng/μl) and the three sgRNAs (100 ng/μl each). Then the embryos ware placed in a line in the gap of an electrode (LF501PT1–10, BEX, Tokyo, Japan) filled with RNA-containing 0.1% PVA-Opti-MEM I (total 5 μl in volume) and electroporation was performed using a CUY21EDIT II electroporator (BEX). After electroporation, the embryos were immediately collected from the electrode chamber and transferred into oviducts of 0.5-day-post-coitum recipients.

### Generation and genotyping and of *Lamp5* KO mice

Genomic DNA was extracted from mouse tail biopsies to serve as templates for genotyping PCR. The PCR primers for the *Lamp5* locus were as follows: Fw, AATATGAGTTTGGTGGCGGT; Rv, TCATTGCATTCGCTCGGTAA. The genotypes of the founder generation were determined by sequencing the amplified PCR fragment directly. One allele of a founder male had a deletion spanning from exon 2 to 6 (Fig. 4a-b), which resulted in truncation of most of the encoded polypeptide (Fig. 4c). This male was mated with C57BL/6J females to obtain F1 mice. For biochemical and behavioral analyses, we used F2 and F3 generation of the mice.

### Antibodies

An anti-LAMP5 polyclonal antibody was raised in rabbit against the peptide “QVQIPRDRSQYKHM”, corresponding to the cytoplasmic tail of LAMP5 (Fig. 4c). We confirmed the specificity of the antibody to LAMP5 by immunoblotting and immunohistochemistry with *Lamp5* KO samples (Fig. 4d and Additional file 1). An anti-LAMP5 guinea pig polyclonal antibody (#412005) was purchased from Synaptic Systems (Goettingen, Germany). This antibody was used only for co-immunostaining of brainstem and spinal cord slices (Fig. 3). An anti-β-actin (#A2228) mouse monoclonal antibody was purchased from Sigma-Aldrich Japan (Tokyo, Japan). An anti-VGAT/VIAAT mouse monoclonal antibody (#131011) was purchased from Synaptic Systems. An anti-VGAT/VIAAT guinea pig polyclonal antibody, and anti-VGluT1 and anti-VGluT2 goat polyclonal antibodies were kindly provided by Dr. Masahiko Watanabe (Hokkaido University). Horseradish peroxidase (HRP)-conjugated anti-rabbit IgG (H + L) (#474–1506) secondary antibody was purchased from KPL (Gaithersburg, MD, USA), and HRP-conjugated anti-rabbit IgG (H + L) (#711–035-152) and anti-mouse IgG (H + L) (#715–035-152) secondary antibodies were from Jackson ImmunoResearch (West Grove, PA, USA). Alexa Fluor 488-labeled donkey anti-rabbit IgG (H + L) (#A21206), goat anti-guinea pig IgG (H + L) (#A11073) and donkey anti-goat IgG (H + L) (#705–035-003) secondary antibodies were purchased from Thermo Fisher Scientific. Cy3-labeled donkey anti-guinea pig IgG (H + L) (#706–165-148) and Cy5-labeled donkey anti-mouse IgG (H + L) (#715–175-151) secondary antibodies were purchased from Jackson ImmunoResearch.

### Western blotting

To analyze LAMP5 expression in brains and spinal cords, brains were dissected from male mice and homogenized using a motor-driven glass Teflon homogenizer in RIPA buffer [50 mM Tris-HCl (pH 8.0), 150 mM NaCl, 1% Triton X-100, 0.5% sodium deoxycholate and 0.1% SDS] supplemented with cOmplete ULTRA (Sigma Aldrich). The homogenate was centrifuged at 13,000×g for 20 min with a tabletop centrifuge. The supernatant was used for further analysis. All protein samples were electrophoretically resolved in 8% gels by SDS-PAGE and electroblotted onto Immobilon-P PVDF membranes (Merck, Darmstadt, Germany). If not otherwise specified, 10 μg of proteins was loaded to each lane. The blots were processed at room temperature by immersion for 1 h in blocking buffer consisting of 5% (*w*/*v*) skimmed milk (Megmilk Snow Brand, Sapporo, Japan) in 1× TBS containing 0.1% (*v*/v) Tween 20 (TBST), then exposing them to primary antibody in blocking buffer for 1 h, washing them with TBST and, finally, exposing them to HRP-conjugated secondary antibody in blocking buffer for 1 h. After washing with TBST, bound antibody was detected using Immobilon Western Chemiluminescent HRP substrate (Merck) or ECL Prime (GE Healthcare Japan, Tokyo, Japan). Images were obtained using ImageQuant LAS 4000 mini (GE Healthcare Japan).

### Reverse transcription PCR

Total RNA was extracted from freshly frozen brain tissues using an RNeasy Mini Kit (QIAGEN, Tokyo, Japan) and reverse transcribed using a PrimeScirpt RT-PCR Kit (Takara Bio, Shiga, Japan) with oligo dT primer. cDNA was amplified with Ex Taq (Takara Bio). The sequences of the primers used in RT-PCR for *Lamp5* and *Gapdh* were as follows: Lamp5-Fw, TCACCATGATCCTGTCCGCA; Lamp5-Rv, CCCATGTGCTTGTACTGGGAA; Gapdh-Fw, CCGTGTTCCTACCCCCAATGTGTCC; Gapdh-Rv, GCCATGTAGGCCATGAGGTCCACCAC. For semi-quantitative analysis, the number of thermal cycles was determined so that the amplification was not saturated. PCR products were electrophoretically resolved on 1% agarose gels and stained with ethidium bromide.

### Immunohistochemistry

Forty-micrometer-thick sagittal sections of male mouse brains were rinsed once with PBS and immersed in blocking buffer [1 × PBS containing 0.2% (v/v) Triton X-100 and 2–5% (v/v) donkey serum (D9663, Sigma-Aldrich)] for 30 min before incubation with primary antibody in blocking buffer for 2 h or overnight at room temperature. The sections were then washed three times with PBS and incubated with secondary antibody for 2 h at room temperature. After three washes with PBS, the sections were mounted using VECTASHIELD Mounting Medium (Vector Laboratories, Burlingame, CA, USA). Images were obtained using a fluorescence microscope (BZ-8000, KEYENCE, Osaka, Japan) and a confocal laser microscope (SP5, Leica microsystems, Wetzlar, Germany). For quantitative analysis of immunoreactivity, images were obtained using a confocal microscope with the pinhole open to be the maximum and were processed using Image J software (v.1.51) [49]. Regions of interest (ROIs) were drawn manually by visual inspection. A rectangular ROI was also chosen in an area where a specimen was not present to serve as a background. Background-subtracted mean gray values for each mouse were pooled and averaged across mice of each genotype. At least three sections per mouse were used.

### Behavioral tests

*Lamp5* KO males and littermate control males were subjected to behavioral tests. Cohort A (*n* = 8 for each genotype) was subjected to open field, elevated plus maze, rotarod and tactile startle response tests in this order at 12 weeks of age. Cohort B (*n* = 5 for each genotype) was subjected to auditory startle response and prepulse inhibition (PPI) tests at 13 weeks of age. All mice were habituated to the testing room for at least 1 h before each experiment.

#### Open field test

Mice were placed in the center of the open field (75 cm diameter) and allowed to explore freely the arena for 5 min under bright light conditions (600 lx). Their movement was recorded via a camera mounted above the arena, and their activity was measured automatically using Smart 3.0 video tracking software (Panlab, Barcelona, Spain). The open field was divided into an inner circle (50 cm diameter), and an outer area surrounding the inner circle. Measurements included total distance traveled and time spent in the inner and outer sections. The open field arena was cleaned with 70% ethanol and wiped with paper towels between each trial.

#### Elevated plus maze test

The elevated plus-maze consisted of two open arms (29.5 cm long × 6 cm wide), two closed arms surrounded by wall (15 cm high) and a central platform (6 cm × 6 cm) halfway between all arms. The entire apparatus was elevated to height of 55 cm above floor level. Mice were placed in the central platform facing an open arm and allowed to move freely through the maze for 10 min under moderate light conditions (25 lx). Their behavior was recorded via a camera mounted above the apparatus. Time spent in the open and closed arms was measured. The maze was cleaned with 70% ethanol and wiped with paper towels between each trial.

#### Rotarod test

The rotarod (Muromachi Kikai, Tokyo, Japan) consisted of a gritted metal roller (3 cm in diameter). A mouse was placed on the rotating roller and the time it remained on the roller was measured. The rotation speed of the roller was initially 4 rpm and increased linearly to 40 rpm over 300 s. One trial was carried out per day and the test was conducted for four consecutive days.

#### Startle response test and PPI test

The startle response was measured using an SR-Lab system (San Diego Instruments, San Diego, CA, USA). A mouse was placed in a small/medium animal enclosure (12.7 cm, 3.8 cm inner diameter) under moderately bright light conditions (260 lx) and was allowed to acclimate for 10 min. The movement of the animal in the startle chamber was measured by a piezoelectric accelerometer mounted under the enclosure at the sampling rate of 1 kHz. For the tactile startle response test, animals were subjected to 20 startle trials and five no-stimulus trials. In each startle trial, animals received an air-puff at the pressure of 0.1 MPa from a tube installed through a hole on the top of the enclosure. The puff of air lasted 100 msec. During the session, including the acclimation period, background white noise of 73 dB was continually present to reduce responses induced by noise from the air tubing. For the auditory startle response and PPI test, the animal then received 20 startle trials, 10 no-stimulus trials and 40 PPI trials. The inter-trial intervals were between 10 and 20 s and the total session lasted 17 min. The startle trial consisted of a single 120 dB white noise burst lasting 40 ms. The PPI trial consisted of a prepulse (20 ms burst of white noise at 69, 73, 77 or 81 dB intensity) followed, 100 ms later, by the startle stimulus (120 dB, 40 ms white noise). Each of the four prepulse trials (69, 73, 77 or 81 dB) was presented 10 times. Five consecutive startle trials were presented at the beginning and end of the session. The remainder of sixty different trials was presented pseudorandomly, ensuring that each trial was presented 10 times and that no two consecutive trials were identical. During the session, including the acclimation period, background white noise of 65 dB was continually present. The recording started 20 ms after startle stimulus onset and lasted for 60 ms. The largest amplitude in the recording window was taken as the startle amplitude for the trial. Basal startle amplitude was determined as the mean startle amplitude of the 20 startle trials. PPI (%) was calculated as follows: 100 × (pulse-alone response – prepulse-pulse response)/pulse-alone response, in which the prepulse-pulse response was the mean of the 10 PPI trials (69, 73, 77 or 81 dB) and the pulse-alone response was the basal startle amplitude.

### Auditory brainstem response

ABR was recorded from mouse cohort A after the series of behavioral analyses as described previously [50]. Briefly, mice were anesthetized with ketamine hydrochloride (50 mg/kg, i.p.) and xylazine hydrochloride (10 mg/kg, i.p.). Before the ABR measurements, the external auditory canals and tympanic membranes were confirmed to be normal in all mice. Subdermal needle electrodes were placed on the vertex (active electrode), in the postauricular area of the measured ear (reference electrode), and in the postauricular area of the opposite ear (ground electrode). ABRs were evoked with tone-bursts (5 ms duration at 4, 8, 16, and 31.25 kHz) and measured by a recording system (Neuropack Ʃ MEB2208, Nihon Kohden). A speaker was placed 10 cm from the tragus of the stimulated ear. ABRs from 500 trials were averaged at each sound intensity. ABR thresholds were estimated by changing the intensity in 5 bB steps and finding the lowest sound intensity where reliable response peaks were detected. Peak amplitudes of ABR waves I, II and V were defined as follows: wave I, height from the baseline (0 ms); wave II, height from the subsequent trough; wave V, height from the previous trough.

## Additional file

Additional file 1: Specificity of the anti-LAMP5 antibody in immunohistochemical staining. (PNG 348 kb)

## Abbreviations

ABR: Auditory brainstem response
CNS: Central nervous system
CRISPR: Clustered regularly interspaced short palindromic repeat
DCO: Dorsal parts of the cochlear nucleus
DNA: Deoxyribonucleic acid
E: Embryonic day
GABA: Gamma-Aminobutyric acid
Gapdh: Glyceraldehyde-3-phosphate dehydrogenase
HRP: Horse raddish peroxidse
KO: Knock out
LAMP: Lysosome-associated membrane protein
LSO: Lateral superior olive
MNTB: Medial nucleus of the trapezoid body
MOCS: Medial olivocochlear system
mRNA: Messenger ribonucleic acid
MSO: Medial superior olive
P: Postnatal day
PAGE: Polyacrylamide gel electrophoresis
PAM: Protospacer adjacent motif
PBS: Phosphate-buffered saline
PCR: Polymerase chain reaction
PnC: Caudal pontine reticular formation
PPI: Prepulse inhibition
PSV: Principal sensory nucleus of the trigeminal
PVA: Polyvinylalchol
PVCN: Posteroventral cochlear nucleus
PVDF: Polyvinylidene difluoride
RNA: Ribonucleic acid
ROI: Region of interest
RT: Reverse transcription
SDS: Sodium dodecyl sulfate
sgRNA: Single guide ribonucleic acid
SNr: Substantia nigra par reticulata
SOC: Superior olivary complex
SPN: Superior paraolivary nucleus
TBS: Tris-buffered saline
VCO: Ventral parts of the cochlear nucleus
VGAT: Vesicular GABA transporter
VGluT: Vesicular glutamate transporter
VIAAT: Vesicular inhibitory amino acid transporter
vNLL: Ventral part of the nucleus of lateral lemniscus
VNTB: Ventral nucleus of the trapezoid body
VP: Ventral pallidum
WT: wild type
