# Supplementary figures and images for: LAMP5 in presynaptic inhibitory terminals in the hindbrain and spinal cord: a role in startle response and auditory processing
Source: Mol Brain. 2019 Mar 12;12:20. doi: 10.1186/s13041-019-0437-4 (PMC6416879; doi:10.1186/s13041-019-0437-4)

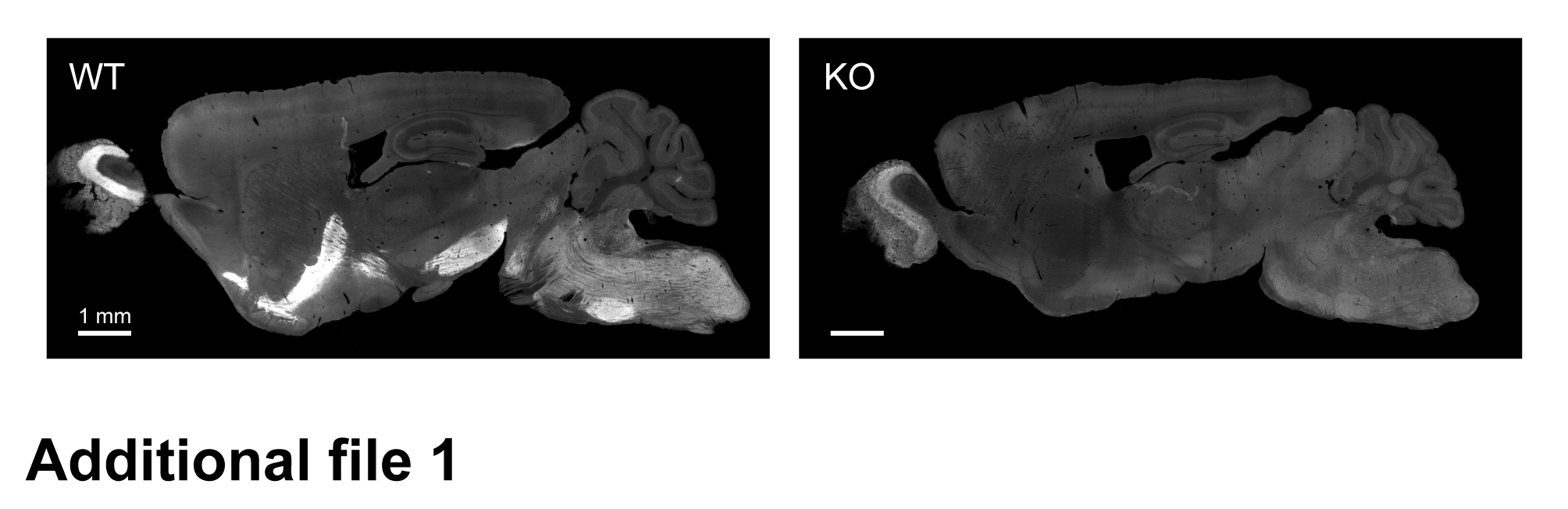

Supplement: Supplementary file 1 — Specificity of the anti-LAMP5 antibody in immunohistochemical staining. (PNG 348 kb) [file 13041_2019_437_MOESM1_ESM.png]
